# SHON expression predicts response and relapse risk of breast cancer patients after anthracycline-based combination chemotherapy or tamoxifen treatment

**DOI:** 10.1038/s41416-019-0405-x

**Published:** 2019-02-28

**Authors:** Tarek M. A. Abdel-Fatah, Reuben J. Broom, Jun Lu, Paul M. Moseley, Baiqu Huang, Lili Li, Suling Liu, Longxin Chen, Runlin Z. Ma, Wenming Cao, Xiaojia Wang, Yan Li, Jo K. Perry, Mohammed Aleskandarany, Christopher C. Nolan, Emad A. Rakha, Peter E. Lobie, Stephen Y. T. Chan, Ian O. Ellis, Le-Ann Hwang, David P. Lane, Andrew R. Green, Dong-Xu Liu

**Affiliations:** 1 Department of Clinical Oncology, University of Nottingham and Nottingham University Hospitals NHS Trust, Nottingham, UK; 2 National Liver Institute, Menoufyia University, Menoufyia, Egypt; 3 Auckland City Hospital, Auckland, New Zealand; 4 The Institute of Genetics and Cytology, Northeast Normal University, Changchun, China; 5 The Key Laboratory of Molecular Epigenetics of Ministry of Education (MOE), Northeast Normal University, Changchun, China; 6 Department of Bone and Soft Tissue Tumors, Tianjin Medical University Cancer Institute and Hospital, Tianjin, China; 7 Fudan University Shanghai Cancer Center & Institutes of Biomedical Sciences, Shanghai Medical College, Key Laboratory of Breast Cancer in Shanghai, Cancer Institutes, Fudan University, Shanghai, China; 8 Laboratory of Molecular Biology, Zhengzhou Normal University, Zhengzhou, China; 9 Institute of Genetics and Developmental Biology, Chinese Academy of Sciences, Beijing, China; 10 Department of Medical Oncology, Zhejiang Cancer Hospital, Hangzhou, China; 11 The Centre for Biomedical and Chemical Sciences, School of Science, Faculty of Health and Environmental Sciences, Auckland University of Technology, Auckland, New Zealand; 12 Liggins Institute, University of Auckland, Auckland, New Zealand; 13 Nottingham Breast Cancer Research Centre, Division of Cancer and Stem Cells, School of Medicine, University of Nottingham, Nottingham City Hospital, Nottingham, UK; 14 Department of Histopathology, School of Medicine, Nottingham University Hospitals NHS Trust, University of Nottingham, Nottingham, UK; 15 Tsinghua Berkeley Shenzhen Institute, Tsinghua University, Shenzhen, Guangdong China; 16 p53 Laboratory, Biomedical Sciences Institutes, Agency for Science, Technology and Research (A*STAR), Singapore, Singapore

**Keywords:** Predictive markers, Molecular medicine, Prognostic markers

## Abstract

**Background:**

SHON nuclear expression (SHON-Nuc^+^) was previously reported to predict clinical outcomes to tamoxifen therapy in ERα^+^ breast cancer (BC). Herein we determined if SHON expression detected by specific monoclonal antibodies could provide a more accurate prediction and serve as a biomarker for anthracycline-based combination chemotherapy (ACT).

**Methods:**

SHON expression was determined by immunohistochemistry in the Nottingham early-stage-BC cohort (*n* = 1,650) who, if eligible, received adjuvant tamoxifen; the Nottingham ERα^−^ early-stage-BC (*n* = 697) patients who received adjuvant ACT; and the Nottingham locally advanced-BC cohort who received pre-operative ACT with/without taxanes (Neo-ACT, *n *= 120) and if eligible, 5-year adjuvant tamoxifen treatment. Prognostic significance of SHON and its relationship with the clinical outcome of treatments were analysed.

**Results:**

As previously reported, SHON-Nuc^+^ in high risk/ERα^+^ patients was significantly associated with a 48% death risk reduction after exclusive adjuvant tamoxifen treatment compared with SHON-Nuc^−^ [HR (95% CI) = 0.52 (0.34–0.78), *p *= 0.002]. Meanwhile, in ERα^−^ patients treated with adjuvant ACT, SHON cytoplasmic expression (SHON-Cyto^+^) was significantly associated with a 50% death risk reduction compared with SHON-Cyto^−^ [HR (95% CI) = 0.50 (0.34–0.73), *p* = 0.0003]. Moreover, in patients received Neo-ACT, SHON-Nuc^−^ or SHON-Cyto^+^ was associated with an increased pathological complete response (pCR) compared with SHON-Nuc^+^ [21 vs 4%; OR (95% CI) = 5.88 (1.28–27.03), *p* = 0.012], or SHON-Cyto^−^ [20.5 vs. 4.5%; OR (95% CI) = 5.43 (1.18–25.03), *p* = 0.017], respectively. After receiving Neo-ACT, patients with SHON-Nuc^+^ had a significantly lower distant relapse risk compared to those with SHON-Nuc^−^ [HR (95% CI) = 0.41 (0.19–0.87), *p* = 0.038], whereas SHON-Cyto^+^ patients had a significantly higher distant relapse risk compared to SHON-Cyto^−^ patients [HR (95% CI) = 4.63 (1.05–20.39), *p *= 0.043]. Furthermore, multivariate Cox regression analyses revealed that SHON-Cyto^+^ was independently associated with a higher risk of distant relapse after Neo-ACT and 5-year tamoxifen treatment [HR (95% CI) = 5.08 (1.13–44.52), *p* = 0.037]. The interaction term between ERα status and SHON-Nuc^+^ (*p* = 0.005), and between SHON-Nuc^+^ and tamoxifen therapy (*p* = 0.007), were both statistically significant.

**Conclusion:**

SHON-Nuce^+^ in tumours predicts response to tamoxifen in ERα^+^ BC while SHON-Cyto^+^ predicts response to ACT.

## Background

Annually there are approximately 2.1 million new cases of female breast cancer (BC) in the world.^1^ Despite improved treatment options, an estimated 626,000 women still die from this disease each year.^1^ BC is not one single disease but consists of a complex group of diseases that are highly heterogeneous in terms of genotype, phenotype, sensitivity to treatment, and clinical outcome.^2^ The success of improved personalised BC therapy relies on the development of robust and accurate biomarkers to guide clinical decision-making in the management of BC.

While targeted therapies are preferable to chemotherapy as first-line treatment for patients with oestrogen receptor α positive (ERα^+^) and HER2-positive (HER2^+^) metastatic BC, chemotherapy is often the initial therapeutic modality of choice for triple negative, and locally advanced or metastatic BC. A meta-analysis of 123 randomised trials involving more than 100,000 patients over 40 years has concluded that standard chemotherapy reduced two-year recurrence rates by 50%, eight-year recurrence rates by approximately one-third, and overall mortality rates by 20–25%.^3^ However, one obstacle to greater success with chemotherapy treatment is drug resistance (acquired or/and intrinsic).^4^ Currently, there is still no definitive methodology to distinguish tumours that will or will not respond to chemotherapies.^5,6^

*SHON* is a recently identified secreted hominoid-specific oncogene in BC.^7^ Forced expression of SHON in BC cell lines significantly increases cell proliferation and survival, promotes anchorage independent growth and enhances cell migration/invasion.^7^ Furthermore, SHON enhances the oncogenicity of BC cells in xenograft models and is sufficient to oncogenically transform MCF10A human normal breast cells.^7^ It has also been shown that SHON regulates epithelial-mesenchymal transition (EMT) through TGF-β signalling in BC cells.^8^ More importantly, *SHON* is an oestrogen inducible gene and its expression in ERα^+^ breast tumours has been shown to be a potential prognostic biomarker for predicting a patient's response to endocrine therapy.^7^ On the other hand, SHON expression is also observed in ERα^−^ BC cell lines such as BT549 and MDA-MB-231, as well as in ERα^−^ BC tissues.^7^ However, the clinical implication of SHON expression in ERα^−^ breast tumours remains unknown.

In the present study, we analysed SHON protein expression in a large cohort of breast tumours by immunohistochemical (IHC) staining using a newly generated anti-SHON monoclonal antibody and determined the relationship of SHON expression with the clinical outcome of chemotherapy-treated patients in another two independent cohorts. We not only validated that SHON nuclear expression in tumour cells was an accurate predictive biomarker for ERα^+^ patients who received tamoxifen, but also identified that SHON cytoplasmic expression in ERα^−^ tumours was able to predict the response of a patient to anthracycline-based treatment.

## Materials and Methods

### The Nottingham University Hospitals early-stage BC cohort

SHON protein expression was examined in a consecutive series of 1,650 patients with primary invasive breast carcinomas who were diagnosed between 1986 and 1999 and entered into the Nottingham University Hospitals (NUH) early-stage BC (NUH-ES-BC) cohort. All patients were treated uniformly in a single institution and have been investigated in a wide range of biomarker studies.^9–11^ Supplementary Table S1 summarises the patient demographics. Patients received standard surgery (mastectomy or wide local excision) with radiotherapy. Prior to 1989, patients did not receive either endocrine therapy or chemotherapy. After 1989, adjuvant therapy was scheduled on the basis of the Nottingham Prognostic Index (NPI), ERα and menopausal status. Patients with NPI scores < 3.4 (low risk) did not receive adjuvant therapy. Pre-menopausal patients with NPI scores ≥ 3.4 (high risk) received Cyclophosphamide, Methotrexate and 5-Fluorouracil (CMF) combination chemotherapy, and patients with ERα^+^ tumour were also received tamoxifen for 5 years. The minimum follow-up period was 123 months and the BC specific survival (BCSS) was used as a primary endpoint.

### The NUH ERα^−^ early-stage BC cohort

In order to assess the value of SHON protein expression as a biomarker in the context of current combination cytotoxic chemotherapy, we also analysed its expression in the NUH ERα^−^ early-stage BC (NUH-ERα^−^ESBC) cohort. It is an independent series of 697 patients who had been diagnosed and managed at the same institution between 1999 and 2007, 141 of whom were treated with adjuvant anthracycline-based combination chemotherapy (ACT). Comprehensive follow-up data were available for 275 patients with BCSS as a primary endpoint (median = 89 months, mean = 86 months; Supplementary Table S1).

### The NUH locally advanced BC cohort

The relationship between SHON protein expression and response to chemotherapy was evaluated by investigating its expression in the pre-chemotherapy core biopsies from 120 female patients with locally advanced (stage IIIA-C) primary BC (NUH-LABC), who were treated with anthracycline-based Neo-ACT (Neo-ACT) at the Nottingham City Hospital between 1996 and 2012. Fifty-three percent (62/120) of the patients received six cycles of anthracycline-based therapy, *i.e*. FEC: 5-Fluorouracil (5-FU) 500 mg m^−2^, Epirubicin 75–100 mg m^−2^, Cyclophosphamide 500 mg m^−2^, on day 1 of a 21-day cycle, and 47% (54/120) of the patients received three cycles of the FEC plus three cycles of taxane (Docetaxel; 100 mg m^−2^). All patients underwent mastectomy or breast-conserving surgery and axillary dissection, followed by adjuvant radiation therapy and if tumours were ERα^+^, 5-year tamoxifen treatment. The median follow-up time was 67 months (IRQ 27–81).

### Survival data

Survival data including survival time, disease-free survival (DFS), and development of loco-regional and distant metastases (DM) were maintained on a prospective basis. DFS was defined as the number of months from diagnosis to the occurrence of recurrence or DM relapse. BCSS was defined as the number of months from diagnosis to the occurrence BC-related death. Survival was censored if the patient was still alive, lost to follow-up, or died from other causes. The study was carried out according to the Reporting Recommendations for Tumour Marker Prognostic Studies (REMARK) criteria.^12^

### Tissue microarrays and immunohistochemistry

Tumours were incorporated into tissue microarrays (TMAs). These were constructed using six replicate 0.6 mm cores from the centre and periphery of the tumours of each patient.

We produced a mouse monoclonal antibody against the mature SHON peptide. The specificity of the mouse anti-SHON monoclonal antibody was determined by Western blot analysis and indirect immunofluorescence staining. The antibody was able to specifically recognise both the endogenous and forced expression of SHON protein in human BC cell lines (Supplementary Figure S1).

The TMAs and full face sections were immunohistochemically profiled with the anti-SHON monoclonal antibody and other antibodies (Supplementary Table S2) using a Novolink Detection kit according to the manufacturer’s protocol (Leica Microsystems, UK) as we previously described.^7^ Sections were pre-treated by boiling in citrate buffer (pH 6.0) for 20 min, and incubated at room temperature for 60 min with the anti-SHON monoclonal antibody at a final concentration of 4 µg/ml. Expression of HER2, ERα and PR was assessed according to the American Society of Clinical Oncology/College of American Pathologists (ASCO/CAP) guidelines.^13,14^

To validate the use of TMAs for immuno-phenotyping, full-face sections of 40 cases were stained and the protein expression levels were compared. The concordance between TMAs and full-face sections was excellent using Cohen's kappa statistical test for categorical variables (kappa = 0.8). Positive and negative (omission of the primary antibody and IgG-matched serum) controls were included in each run.

### Evaluation of SHON IHC staining

**Fig. 1:**
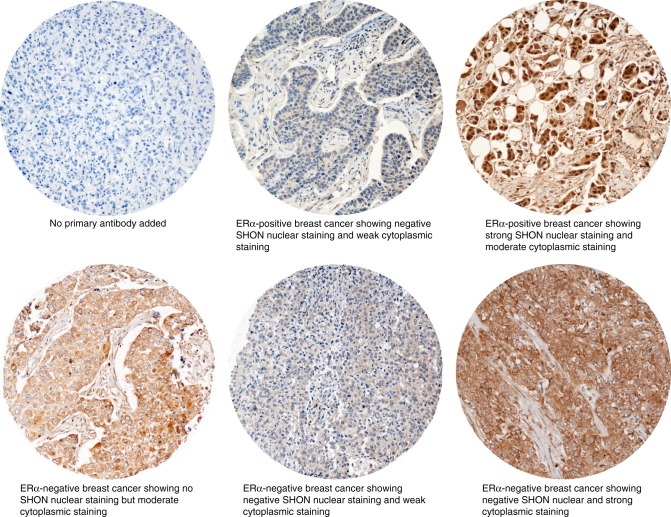
Microphotographs of SHON expression in representative breast cancer TMA cores. SHON expression was determined by IHC using a SHON mouse monoclonal antibody. ERα, oestrogen receptor α

Tumour cores were evaluated by two pathologists who were blinded to the clinicopathological characteristics of patients in two different settings. Whole field inspection of the core was scored, and intensities of both nuclear and cytoplasmic staining were grouped as follows: 0 = no staining, 1 = weak staining, 2 = moderate staining, and 3 = strong staining. The percentage of each category was estimated, and the H-score was calculated as previously described.^9^ Due to intra- and inter-tumoural heterogeneity of staining, the average percentage was calculated. The cut-off of SHON cytoplasmic and nuclear staining was determined by using the median expression. High cytoplasmic staining was defined as the presence of H-score > 150, whereas high nuclear staining was defined as the presence of ≥ 1% positive nuclear staining (Fig. 1). Intra- (kappa > 0.8; Cohen kappa test) and inter- (kappa > 0.8; using multi-rater kappa tests) observer agreements were excellent. In cases where discordant results were obtained, the slides were re-evaluated by both pathologists together and a consensus was reached.

### Statistical analysis

Data analyses were performed using SPSS statistics software (version 17, Chicago, IL). Where appropriate, Pearson’s Chi-square, and Student’s t-test were used. Significance was defined at *p* < 0.05.

Cumulative survival probabilities were estimated using the Kaplan–Meier method, and differences between survival rates were tested for significance using the log-rank test. Multivariate analyses for survival were performed using the Cox proportional hazard model. The proportional hazards assumption was tested using standard log-log plots. Hazard ratios (HR) and 95% confidence intervals (95% CI) were estimated for each variable. All tests were two-sided with a 95% CI, and a *p* value < 0.05 was considered to be indicative of statistical significance. A stringent *p* value < 0.01 was considered to indicate statistical significance for multiple comparisons.

## Results

### Sub-cellular compartmentalisation of SHON protein expression

A total of 1,299 tumours in the NUH-ES-BC cohort were suitable for the IHC analysis of SHON protein expression. High nuclear SHON (SHON-Nuc^**+**^) staining was observed in 205/1,299 (16%) tumours compared to 1,094/1,299 (84%) tumours that had no nuclear SHON staining (SHON-Nuc^−^). However, 865/1,299 (67%) tumours exhibited high cytoplasmic staining (SHON-Cyto^**+**^) compared with 434/1,299 (33%) tumours that had low cytoplasmic expression (SHON-Cyto^−^). There was an inverse correlation between cytoplasmic and nuclear SHON expression (*p* < 0.0001). The majority of tumours (766/1,299; 59%) were SHON-Cyto^**+**^**/**Nuc^**−**^ phenotype. The percentages of SHON-Cyto^−^**/**Nuc^−^, SHON-Cyto^−^/Nuc^+^ and SHON-Cyto^+^/Nuc^+^ tumours were 25% (328/1,299), 8% (106/1,299) and 8% (99/1,299), respectively.

### Association of SHON nuclear expression with favourable clinicopathological characteristics

SHON nuclear expression was associated with favourable clinicopathological features including hormone receptor (ERα^+^, PR^+^ and AR^+^) positivity, 4-IHC luminal A (ERα^+^/HER2^−^/low proliferation phenotype), tubular BC, low histological grade, low mitotic index, low proliferation index (Ki67), low pleomorphism, and MDM4 overexpression (Table 1). Furthermore, SHON-Nuc^**+**^ was highly associated with high expression of DNA repair proteins: PARP1, TOPO2A, RECQL4 Nuclear, RECQL5, BLM Nuclear, CHK1, CHK2, and Phosphorylated CHK1 Nuclear (Table 1).

**Table 1:** Association between SHON protein nuclear expression and clinicopathological variables in the NUH-ES-BC cohort (*n *= 1,650)

Variables	SHON protein nuclear expression		*χ*^*2*^ *p* value (2 sided)
	Low *N* (%)	High *N* (%)	
(A) Pathological parameters			
Lymph node (LN) metastases			0.824
Negative	753 (62.0)	28 (63.6)	
Positive	462 (38.0)	16 (36.4)	
Grade^a^			<0.001*
Low (G1)	183 (15.1)	17 (38.6)	
Intermediate (G2)	373 (30.8)	20 (45.5)	
High (G3)	656 (54.1)	7 (15.9)	
Tumour size (cm)			0.332
T1 a + b (≤ 1.0)	120 (9.9)	6 (13.6)	
T1 c (> 1.0–2.0)	596 (49.2)	26 (59.1)	
T2 (> 2.0–5.0)	462 (38.1)	11 (25.0)	
T3 (> 5.0)	34 (2.8)	1 (2.3)	
Mitotic index			<0.001*
M1	370 (30.8)	26 (61.9)	
M2	231 (19.2)	8 (19.0)	
M3	600 (50.0)	8 (19.0)	
Pleomorphism			<0.001*
P1	24 (2.0)	1 (2.4)	
P2	428 (35.6)	29 (69.0)	
P3	749 (62.4)	12 (28.6)	
Tubule formation			0.004*
T1	68 (5.7)	2 (4.8)	
T2	394 (32.8)	24 (57.1)	
T3	739 (61.5)	16 (38.1)	
Lympho-vascular invasion			0.587
Positive	788 (65.8)	30 (69.8)	
Negative	410 (34.2)	13 (30.2)	
Histological type of invasive carcinoma			0.016*
Invasive ductal carcinoma - no special type	637 (61.5)	11 (36.7)	
Tubular carcinoma	210 (20.3)	8 (26.7)	
Medullary carcinoma	25 (2.4)	0 (0.0)	
Invasive lobular carcinoma	79 (7.6)	5 (16.7)	
Others	84 (8.1)	6 (20.0)	
(B) Molecular characteristics			
ERα (IHC)			0.044*
Negative	348 (29.1)	6 (14.6)	
Positive	848 (70.9)	35 (85.4)	
PR (IHC)			0.049*
Negative	507 (45.1)	11 (28.9)	
Positive	617 (54.9)	27 (71.1)	
HER2 overexpression			0.052
No	1,038 (87.7)	41 (97.6)	
Yes	145 (12.3)	1 (2.4)	
HER3 (IHC)			0.155
Negative	474 (49.6)	16 (64.0)	
Positive	482 (50.4)	9 (36.0)	
HER4 (IHC)			0.006*
Negative	401 (41.6)	19 (67.9)	
Positive	563 (58.4)	9 (32.1)	
Androgen receptor (IHC)			0.034*
Negative	369 (39.1)	4 (17.4)	
Positive	574 (60.9)	19 (82.6)	
EGFR (IHC)			0.974
Low	746 (79.7)	16 (80.0)	
High	190 (20.3)	4 (20.0)	
MIB1 (Ki67) (IHC)			0.001*
Low	325 (32.4)	20 (58.8)	
High	679 (67.6)	14 (41.2)	
BRCA1 (IHC)			0.102
Absent	174 (20.4)	1 (5.3)	
Normal	677 (79.6)	18 (94.7)	
SPAG5 (IHC)			0.093
Low	696 (78.7)	24 (92.3)	
High	188 (21.3)	2 (7.7)	
KIF2C (IHC)			<0.001*
Low	264 (32.5)	17 (68.0)	
High	549 (67.5)	8 (32.0)	
PARP1 (IHC)			0.012*
Low	537 (73.5)	9 (47.4)	
High	194 (26.5)	10 (52.6)	
TOPO2A (IHC)			0.001*
Low	398 (46.7)	3 (12.0)	
High	454 (53.3)	22 (88.0)	
P53 (IHC)			0.306
Low	754 (78.1)	20 (87.0)	
High	212 (21.9)	3 (13.0)	
P27 (IHC)			0.057
Low	444 (61.1)	9 (40.9)	
High	283 (38.9)	13 (59.1)	
Cyclin B2 (IHC)			<0.001*
Low	532 (44.2)	31 (72.1)	
High	671 (55.8)	12 (27.9)	
MDM2 (IHC)			0.402
Low	628 (75.3)	12 (66.7)	
High	206 (24.7)	6 (33.3)	
MDM4 (IHC)			0.017*
Low	667 (62.9)	14 (42.4)	
High	393 (37.1)	19 (57.6)	
P21 (IHC)			0.312
Negative	474 (55.6)	14 (66.7)	
Positive	379 (44.4	7 (33.3)	
P16 (IHC)			0.745
Low	686 (84.4)	18 (81.8)	
High	127 (15.6)	4 (18.2)	
P63 (IHC)			0.438
Negative	978 (97.9)	28 (100.0)	
Positive	21 (2.1)	0 (0.0)	
CDK1 (IHC)			0.039*
Low	506 (70.0)	10 (100)	
High	217 (30.0)	0 (0.0)	
BCL-2 (IHC)			0.044*
Low	385 (36.0)	6 (18.8)	
High	683 (64.0)	26 (81.3)	
BAX (IHC)			0.451
Low	465 (69.6)	13 (61.9)	
High	203 (30.4)	8 (38.1)	
CK18 (IHC)			0.663
Negative	108 (11.6)	2 (8.7)	
Positive	820 (88.4)	21 (91.3)	
CK19 (IHC)			0.192
Negative	62 (6.2)	0 (0.0)	
Positive	644 (93.8)	26 (100.0)	
CK14 (IHC)			0.012*
Negative	875 (87.1)	19 (70.4)	
Positive	130 (12.9)	8 (29.6)	
CK6 (IHC)			0.096
Negative	838 (82.7)	19 (70.4)	
Positive	175 (17.3)	8 (29.6)	
SMA (IHC)			0.991
Negative	846 (85.1)	23 (85.2)	
Positive	148 (14.9)	4 (14.8)	
ERCC1 (IHC)			0.007*
Low	344 (61.2)	4 (26.7)	
High	218 (38.8	11 (73.3)	
TDK (IHC)			0.948
Low	461 (59.4)	18 (60.0)	
High	315 (40.6)	12 (40.0)	
RECQL4 cytoplasm (IHC)			0.137
Low	122 (15.2)	7 (25.9)	
High	673 (84.7)	20 (74.1)	
RECQL4 nuclear (IHC)			0.003*
Low	405 (50.9)	6 (22.2)	
High	390 (49.1)	21 (77.8)	
RECQL5 (IHC)			0.027*
Low	429 (47.9)	9 (28.1)	
High	466 (52.1)	23 (71.9)	
Vimentin (IHC)			0.686
Low	920 (88.6)	25 (86.2)	
High	118 (11.4)	4 (13.8)	
E-cadherin (IHC)			0.747
Negative	54 (5.5)	1 (4.0)	
Positive	931 (94.5)	24 (96.0)	
BLM cytoplasm (IHC)			0.533
Low	418 (45.0)	20 (50.0)	
High	511 (55.0)	20 (50.0)	
BLM nuclear (IHC)			0.001*
Low	518 (55.8)	12 (30.0)	
High	411 (44.2)	28 (70.0)	
CHK1 (IHC)			0.016*
Low	504 (52.5)	7 (28.0)	
High	456 (47.5)	18 (72.0)	
ATM cytoplasm (IHC)			0.311
Low	392 (53.2)	6 (40.0)	
High	345 (46.8)	9 (60.0)	
ATR (IHC)			0.098
Low	623 (64.4)	28 (77.8)	
High	345 (35.6)	8 (22.2)	
CHK2 (IHC)			0.039*
Low	389 (48.3)	5 (25.0)	
High	416 (51.7)	15 (75.0)	
Phosphorylated CHK1 nuclear (IHC)			<0.001*
Low	975 (85.9	17 (38.6)	
High	160 (14.1	27 (61.4)	
Phosphorylated CHK1 cytoplasm (IHC)			0.328
Low	359 (31.6)	17 (38.6)	
High	776 (68.4)	27 (61.4)	
XRCC1 (IHC)			0.122
Low	142 (16.3)	1 (4.3)	
High	728 (83.7)	22 (95.7)	
DNA polymerase beta (IHC)			0.036*
Low	396 (39.3)	7 (21.2)	
High	611 (60.7)	26 (78.8)	
DNA PK (IHC)			0.511
Low	317 (35.8)	8 (29.6)	
High	569 (64.2)	19 (70.4)	
SMUG1 (IHC)			0.063
Low	316 (40.7)	4 (20.0)	
High	461 (59.3)	16 (80.0)	
APE1 (IHC)			0.008*
Low	493 (52.1)	9 (28.1)	
High	454 (47.9)	23 (71.9)	
FEN1 (IHC)			<0.001*
Low	606 (74.1)	8 (36.4)	
High	212 (25.9)	14 (63.6)	
Phosphorylated c-Jun (IHC)			0.023*
Low	439 (46.7)	7 (25.0)	
High	501 (53.3)	21 (75.0)	
Phosphorylated JNK (IHC)			0.001*
Low	661 (72.2)	9 (39.1)	
High	255 (27.8)	14 (60.9)	
Phosphorylated p38 (IHC)			0.062
Low	741 (84.1)	16 (69.6)	
High	140 (15.9)	7 (30.4)	
SRC3 (IHC)			0.603
Low	541 (57.2)	15 (62.5)	
High	405 (42.8)	9 (37.5)	
S543 (IHC)			0.001*
Low	727 (82.9)	12 (54.5)	
High	150 (17.1)	10 (45.5)	
ATF2 (IHC)			0.786
Low	455 (49.2)	13 (52.0)	
High	469 (50.8)	12 (48.0)	
T24 (IHC)			0.669
Low	612 (74.6)	15 (78.9)	
High	208 (25.4)	4 (21.1)	
T71 (IHC)			0.252
Low	502 (50.6)	12 (40.0)	
High	490 (49.4)	18 (60.0)	
HAGE (IHC)			0.476
Negative	982 (90.8)	33 (94.3)	
Positive	100 (9.2)	2 (5.7)	
TROAP (IHC)			0.455
Negative	431 (55.7)	11 (47.8)	
Positive	343 (44.3)	12 (52.2)	
Breast cancer sub-groups			0.001*
Luminal A	348 (34.5)	24 (72.7)	
Luminal B (Ki67 ≥15)	314 (31.1)	4 (12.1)	
Luminal B (HER2^+^)	63 (6.2)	0 (0.0)	
Non-luminal HER2^+^	81 (8.0)	1 (3.0)	
Basal-like	155 (15.4)	4 (12.1)	
ERα^−^/HER2^−^ none basal	48 (4.8)	0 (0.0)	
Basal-like phenotype			0.508
No	981 (86.4)	36 (90.0)	
Yes	155 (13.6)	4 (10.0)	
Triple negative phenotype			0.105
No	937 (79.5)	36 (90.0)	
Yes	241 (20.5)	4 (10.0)	

*ERα* oestrogen receptor α, *PR* progesterone receptor, *HER2* human epidermal growth factor receptor 2, *Triple negative* ERα^−^/PR^−^/HER2^−^

*Statistically significant at *p* < 0.05

^a^Grade as defined by the Nottingham Grading System (NGS)

### Association of SHON cytoplasmic expression with aggressive clinicopathological characteristics

SHON cytoplasmic expression was associated with aggressive clinicopathological features including absence of hormone receptor (ERα^−^ and PR^−^) positivity, basal-like phenotype, ERα^−^/HER2^−^, triple negative, invasive ductal carcinoma of no specific type (IDC-NST), higher histological grade, tubular dedifferentiation, pleomorphism, high mitotic index, and higher levels of proliferation markers (all *p* < 0.01) (Table 2).

**Table 2:** Association between SHON protein cytoplasmic expression and clinicopathological variables in the NUH-ES-BC cohort (*n* = 1,650)

Variables	SHON protein cytoplasmic expression		*χ*^*2*^ *p* value (2 sided)
	Low *N* (%)	High *N* (%)	
(A) Pathological parameters			
Lymph node (LN) metastases			0.432
Negative	343 (63.2)	457 (61.0)	
Positive	200 (36.8)	292 (39.0)	
Grade^a^			<0.001*
Low (G1)	102 (18.8)	106 (14.2)	
Intermediate (G2)	192 (35.3)	213 (28.6)	
High (G3)	250 (46.0)	426 (57.2)	
Tumour size (cm)			0.180
T1 a + b (≤ 1.0)	49 (9.0)	80 (10.7)	
T1 c (> 1.0–2.0)	286 (52.6)	286 (38.4)	
T2 (> 2.0–5.0)	198 (36.4)	25 (3.4)	
T3 (> 5.0)	11 (2.0)	25 (3.4)	
Mitotic index			<0.001*
M1	208 (38.9)	204 (27.6)	
M2	100 (18.7)	143 (19.3)	
M3	227 (42.4)	393 (53.1)	
Pleomorphism			<0.001*
P1	13 (2.4)	15 (2.0)	
P2	229 (42.8)	240 (32.4)	
P3	293 (54.8)	485 (65.5)	
Tubule formation			0.488
T1	31 (5.8)	43 (5.8)	
T2	189 (35.3)	238 (32.2)	
T3	315 (58.9)	459 (62.0)	
Lympho-vascular invasion			0.114
Positive	368 (68.8)	477 (64.5)	
Negative	167 (31.2)	262 (35.5)	
Histological type of invasive carcinoma			<0.001*
Invasive ductal carcinoma - no special type	256 (56.6)	409 (63.7)	
Tubular carcinoma	86 (19.0)	138 (21.5)	
Medullary carcinoma	10 (2.2)	15 (2.3)	
Invasive lobular carcinoma	56 (12.4)	32 (5.0)	
Others	44 (9.7)	48 (7.5)	
(B) Molecular characteristics			
ERα (IHC)			<0.001*
Negative	117 (21.8)	224 (33.2)	
Positive	419 (78.2)	490 (66.8)	
PR (IHC)			0.048*
Negative	210 (41.3)	322 (47.0)	
Positive	299 (58.7)	363 (55.4)	
HER2 overexpression			0.008*
No	485 (91.0)	624 (86.1)	
Yes	48 (9.0)	101 (13.9)	
HER3 (IHC)			<0.001*
Negative	236 (56.7)	263 (44.9)	
Positive	180 (43.3)	323 (55.1)	
HER4 (IHC)			0.006*
Negative	204 (47.3)	227 (38.7)	
Positive	227 (52.7)	360 (61.3)	
Androgen receptor (IHC)			0.580
Negative	156 (37.7)	229 (39.4)	
Positive	258 (62.3)	352 (60.6)	
EGFR (IHC)			0.005*
Low	335 (83.8)	442 (76.3)	
High	65 (16.3)	137 (23.7)	
MIB1 (Ki67) (IHC)			0.004*
Low	170 (38.7)	189 (30.1)	
High	269 (61.3)	438 (69.9)	
BRCA1 (IHC)			0.207
Absent	65 (18.1)	114 (21.5)	
Normal	295 (81.9)	416 (78.5)	
SHON nuclear (IHC)			<0.001*
Negative	491 (93.3)	731 (98.8)	
Positive	35 (6.7)	9 (1.2)	
SPAG5 (IHC)			0.03*
Low	321 (82.5)	417 (76.7)	
High	68 (17.5)	127 (23.3	
KIF2C (IHC)			0.003*
Low	138 (39.9)	155 (30.2)	
High	208 (60.1)	358 (69.8)	
PARP1 (IHC)			0.008*
Low	245 (77.8)	314 (69.2)	
High	70 (22.2)	140 (ki67	
TOPO2A (IHC)			0.360
Low	174 (47.5)	236 (44.4)	
High	192 (52.5)	295 (55.6)	
P53 (IHC)			0.121
Low	338 (80.9)	460 (76.8)	
High	80 (19.1)	139 (23.2)	
P27 (IHC)			0.997
Low	198 (61.1)	227 (61.1)	
High	126 (38.9)	173 (38.9)	
Cyclin B2 (IHC)			<0.001*
Low	288 (54.3)	295 (39.4)	
High	242 (45.7)	453 (60.6)	
MDM2 (IHC)			<0.001*
Low	241 (68.7)	412 (79.2)	
High	110 (31.3)	108 (20.8)	
MDM4 (IHC)			0.002*
Low	314 (68.0)	386 (58.8)	
High	148 (32.0)	271 (41.2)	
P21 (IHC)			0.064
Negative	218 (59.7)	284 (53.5)	
Positive	147 (40.3)	247 (46.5)	
P16 (IHC)			0.274
Low	292 (86.1)	431 (83.4)	
High	47 (13.9)	86 (16.6)	
P63 (IHC)			0.925
Negative	433 (98.0)	602 (98.0)	
Positive	9 (2.0)	12 (2.0)	
CDK1 (IHC)			<0.001*
Low	219 (77.9)	307 (65.7)	
High	62 (22.1)	160 (34.3)	
BCL-2 (IHC)			0.384
Low	159 (33.9)	240 (36.4)	
High	310 (66.1)	419 (63.6)	
BAX (IHC)			0.01*
Low	209 (74.9)	282 (65.7)	
High	70 (25.1)	147 (34.3)	
CK18 (IHC)			0.77
Negative	48 (11.9)	85 (11.3)	
Positive	355 (88.1)	510 (88.7)	
CK19 (IHC)			0.308
Negative	31 (7.0)	34 (5.5)	
Positive	411 (93.0)	585 (4.5)	
CK14 (IHC)			0.384
Negative	385 (87.7)	534 (85.9)	
Positive	54 (12.3)	88 (14.1)	
CK6 (IHC)			0.039*
Negative	379 (85.2)	501 (80.3)	
Positive	66 (14.8)	123 (19.7)	
SMA (IHC)			0.036*
Negative	385 (87.5)	505 (82.8)	
Positive	55 (12.5)	105 (17.2)	
ERCC1 (IHC)			0.081
Low	151 (64.5)	200 (57.3)	
High	83 (35.5)	149 (42.7)	
TDK (IHC)			0.407
Low	211 (61.2)	278 (58.3)	
High	134 (38.8)	199 (41.7)	
RECQL4 cytoplasm (IHC)			<0.001*
Low	81 (24.3)	51 (10.1)	
High	252 (75.7)	453 (89.9)	
RECQL4 nuclear (IHC)			0.921
Low	167 (50.2)	251 (49.8)	
High	166 (49.8)	253 (50.2)	
RECQL5 (IHC)			0.023*
Low	204 (51.4)	243 (43.9)	
High	193 (48.6)	310 (56.1)	
Vimentin (IHC)			0.637
Low	400 (89.1)	566 (88.2)	
High	49 (10.9)	76 (11.8)	
E-cadherin (IHC)			0.223
Negative	28 (6.5)	29 (4.8)	
Positive	402 (93.5)	580 (95.2)	
BLM cytoplasm (IHC)			<0.001*
Low	228 (53.6)	219 (38.8)	
High	197 (46.4)	345 (61.2)	
BLM nuclear (IHC)			0.720
Low	231 (54.4)	313 (55.5)	
High	194 (45.6)	251 (44.5)	
CHK1 (IHC)			0.210
Low	219 (53.9)	300 (49.9)	
High	187 (46.1)	301 (50.1)	
ATM cytoplasm (IHC)			0.922
Low	166 (52.7)	243 (53.1)	
High	149 (47.3)	215 (46.9)	
ATR (IHC)			0.011*
Low	294 (69.7)	373 (62.0)	
High	128 (30.3)	229 (38.0)	
CHK2 (IHC)			<0.001*
Low	187 (55.3)	215 (42.6)	
High	151 (44.7	290 (57.4	
Phosphorylated CHK1 nuclear (IHC)			0.217
Low	433 (85.7)	586 (83.1)	
High	72 (14.3)	119 (16.9)	
Phosphorylated CHK1 cytoplasm (IHC)			<0.001*
Low	215 (42.6)	174 (24.7)	
High	290 (57.4)	531 (75.3)	
XRCC1 (IHC)			0.546
Low	64 (16.8)	82 (15.4)	
High	316 (83.2)	452 (84.6)	
DNA polymerase beta (IHC)			<0.001*
Low	201 (45.4)	213 (34.2)	
High	242 (54.6)	409 (65.8)	
DNA PK (IHC)			<0.001*
Low	176 (46.0)	154 (28.1)	
High	207 (54.0)	394 (71.9)	
SMUG1 (IHC)			0.095
Low	124 (36.6)	203 (42.4)	
High	215 (63.4)	276 (57.6)	
APE1 (IHC)			<0.001*
Low	254 (61.5)	260 (44.3)	
High	159 (38.5)	327 (55.7)	
FEN1 (IHC)			0.780
Low	261 (73.7)	368 (72.9)	
High	93 (26.3)	137 (27.1)	
Phosphorylated c-Jun (IHC)			0.023*
Low	209 (50.9)	253 (43.5)	
High	202 (49.1)	328 (56.5)	
Phosphorylated JNK (IHC)			0.280
Low	294 (73.3)	392 (70.1)	
High	107 (26.7)	167 (29.9)	
Phosphorylated p38 (IHC)			0.563
Low	322 (85.0)	457 (83.5)	
High	57 (15.0)	90 (16.5)	
SRC3 (IHC)			0.08
Low	249 (60.6)	319 (55.0)	
High	162 (39.4)	261 (45.0)	
S543 (IHC)			0.866
Low	310 (82.2)	448 (82.7)	
High	67 (17.8)	94 (17.3)	
ATF2 (IHC)			0.325
Low	204 (51.4)	277 (48.2)	
High	193 (48.6)	298 (51.8)	
T24 (IHC)			0.885
Low	261 (75.4)	384 (75.0)	
High	85 (24.6)	128 (25.0)	
T71 (IHC)			0.015*
Low	237 (55.0)	293 (47.3)	
High	194 (45.0)	326 (52.7)	
HAGE (IHC)			0.949
Negative	440 (90.9)	602 (90.8)	
Positive	44 (9.1)	61 (9.2)	
TROAP (IHC)			0.001*
Negative	216 (62.8)	241 (50.6)	
Positive	128 (37.2)	235 (49.4)	
Breast cancer sub-groups			0.001*
Luminal A	184 (41.2)	202 (32.4)	
Luminal B (Ki67 ≥ 15)	142 (31.8)	181 (29.1)	
Luminal B (HER2^+^)	27 (6.0)	38 (6.1)	
Non-luminal HER2^+^	21 (4.7)	62 (10.0)	
Basal like	52 (11.6)	111 (17.8)	
ERα^−^/HER2^−^ none basal	21 (4.7)	29 (4.7)	
Basal-like phenotype			0.003*
No	463 (89.9)	583 (84.0)	
Yes	52 (10.1)	111 (16.0)	
Triple negative phenotype			0.005*
No	441 (83.7)	559 (77.2)	
Yes	68 (16.3)	165 (22.8)	

*ERα* oestrogen receptor α, *PR* progesterone receptor, *HER2* human epidermal growth factor receptor 2, *Triple negative* ERα^−^/PR^−^/HER2^−^

*Statistically significant at *p* < 0.05

^a^Grade as defined by the Nottingham Grading System (NGS)

SHON-Cyto^**+**^**/**Nuc^−^ phenotype exhibited the most aggressive features including absence of hormone receptor (ERα^−^, PR^−^ and AR^−^) positivity, triple negative, basal-like, large size, high stage, high grade, high lymphovascular invasion, overexpression of HER family (HER1^+^, HER2^+^, HER3^+^ and HER4^+^), p53 mutation, dysregulation of both DNA repair and high vimentin.

### SHON protein nuclear expression predicted favourable clinical outcomes of ERα^+^ BC treated with endocrine therapy

**Fig. 2:**
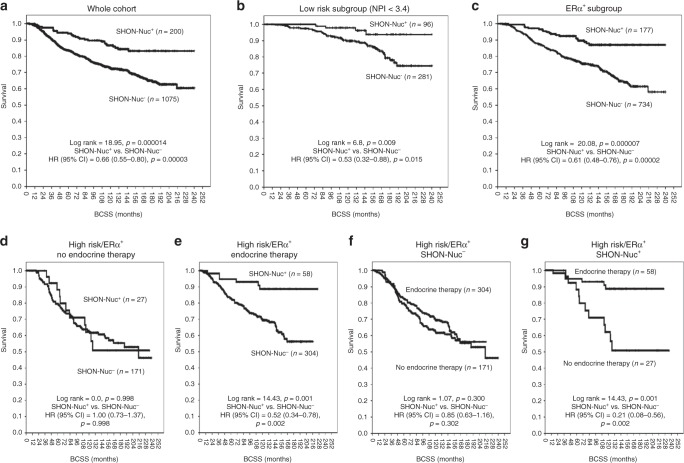
Clinical outcome of SHON protein nuclear expression in breast cancer. Kaplan–Meier plots of the rates of breast cancer specific survival (BCSS; months) in the NUH-ES-BC cohort (*n* = 1,650) according to SHON protein nuclear expression (SHON-Nuc) status. The *p* value from the log rank test is shown in each panel; ‘*n*' is the number of samples in each group. High risk, NPI scores ≥ 3.4; ERα, oestrogen receptor α; +, positive expression; −, negative expression

SHON-Nuc^**+**^ in the whole NUH-ES-BC cohort was associated with prolonged BCSS and a reduced risk of death from BC [HR (95% CI) = 0.66 (0.55–0.80), *p* < 0.0001] (Fig. 2a), in the low risk patients [NPI < 3.4; HR (95% CI) = 0.53 (0.32–0.88), *p* = 0.015] (Fig. 2b), and in the ERα^+^ subgroup [HR (95% CI) = 0.61 (0.48-0.76), *p* < 0.0001] (Fig. 2c).

In high risk (NPI ≥ 3.4)/ERα^+^ patients who did not receive tamoxifen treatment, tumours with or without SHON nuclear protein expression had a similar BCSS rate [HR (95% CI) = 1.00 (0.73–1.37), *p* = 0.998] (Fig. 2d). Meanwhile, SHON nuclear protein expression positivity was very significantly associated with better survival and a 48% lower risk of death in tamoxifen-treated patients [HR (95% CI) = 0.52 (0.34–0.78), *p* = 0.002] compared with SHON nuclear protein expression negativity (Fig. 2e). In high risk/ERα^+^ subgroups, if the tumours were SHON-Nuc^**−**^, administration of tamoxifen had no impact on the survival [HR (95% CI) = 0.85 (0.63–1.16), *p* = 0.302] (Fig. 2f), whereas if the tumours were also SHON-Nuc^**+**^, tamoxifen treatment resulted in improved survival and a reduced risk of death from BC by 79% [HR (95% CI) = 0.21 (0.08–0.56), *p *= 0.002] (Fig. 2g). This result is consistent with our previous observation that SHON nuclear protein expression is a predictor of patient response to tamoxifen treatment in BC.^7^

### SHON protein cytoplasmic expression predicted worse clinical outcomes of BC

**Fig. 3:**
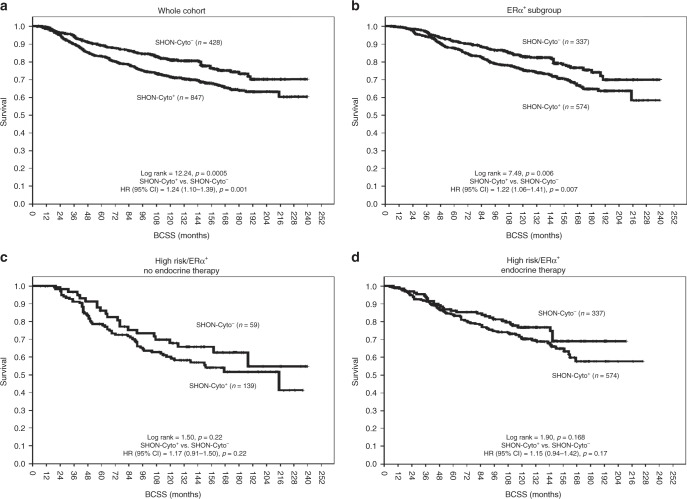
Clinical outcome of SHON protein cytoplasmic expression in breast cancer. Kaplan–Meier plots of the rates of breast cancer specific survival (BCSS; months) in the NUH-ES-BC cohort (*n *= 1,650) according to SHON protein cytoplasmic expression (SHON-Cyto) status. The *p* value from the log rank test is shown in each panel; ‘*n*' is the number of samples in each group. High risk, NPI scores ≥ 3.4; ERα, oestrogen receptor α; +, positive expression; −, negative expression

SHON-Cyto^**+**^ in the whole NUH-ES-BC cohort was associated with shorter BCSS and an increased risk of death from BC [HR (95% CI) = 1.24 (1.10–1.39), *p *= 0.001] (Fig. 3a), and the ERα^+^ subgroup [HR (95% CI) = 1.22 (1.06–1.41), *p *= 0.007] (Fig. 3b). However, there was no association between the impact of tamoxifen on patient survival and SHON cytoplasmic expression in the ERα^+^ subgroup (Fig. 3c, d).

### SHON protein cytoplasmic expression predicted clinical outcomes of ERα^−^ BC treated with anthracycline-based chemotherapy

**Fig. 4:**
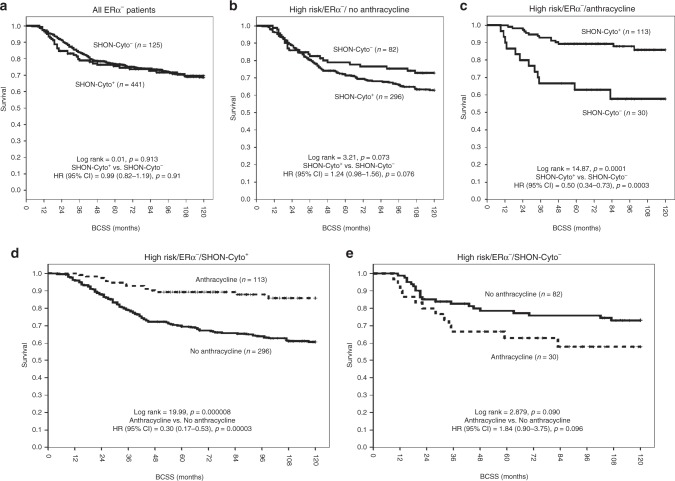
Clinical outcome of SHON protein cytoplasmic expression in ERα^**−**^ breast cancer patients. Kaplan–Meier plots of the rates of breast cancer specific survival (BCSS; months) in the NUH-ERα^−^ESBC cohort (*n *= 697) according to SHON protein cytoplasmic expression (SHON-Cyto) status. The *p* value from the log rank test is shown in each panel; ‘*n*' is the number of samples in each group. High risk, NPI scores ≥ 3.4; ERα, oestrogen receptor α; +, positive expression; −, negative expression

In the ERα^−^ BC subgroup, there was no association between SHON-Cyto^+^ and clinical outcomes in the NUH-ERα^−^ESBC cohort [HR (95% CI) = 0.99 (0.82–1.19), *p* = 0.91] (Fig. 4a). However, SHON cytoplasmic expression predicted better BCSS in those patients who received anthracycline-based combination chemotherapy. As shown in Fig. 4b, SHON-Cyto^+^ was associated with a trend of shorter survival in ERα^−^ patients who did not receive any chemotherapy, though it was not statistically significant [HR (95% CI) = 1.24 (0.98–1.56), *p* = 0.076]. In contrast, in anthracycline-based combination-treated patients with ERα^−^ tumours, SHON-Cyto^**+**^ was highly significantly associated with better BCSS and a lower risk of death compared with SHON-Cyto^**−**^ [HR (95% CI) = 0.50 (0.34–0.73), *p* = 0.0003] (Fig. 4c). Exposure to anthracycline resulted in improved BCSS and a reduced risk of death from BC in tumours with SHON-Cyto^**+**^ [HR (95% CI) = 0.30 (0.17–0.53), *p* = 0.00003] (Fig. 4d), whereas in those with SHON-Cyto^−^, exposure to anthracycline was associated with a trend of shorter survival and a higher risk of death, though it was not statistically significant [HR (95% CI) = 1.84 (0.90–3.75), *p* = 0.096] (Fig. 4e). The interaction term between SHON-Cyto expression and anthracycline chemotherapy was highly significant (*p* < 0.001). These results indicate that SHON cytoplasmic protein expression was able to predict the BCSS of patients with ERα^−^ tumours treated with anthracycline-based chemotherapy.

### The relationship between SHON protein expression and distant relapse risks after receiving Neo-ACT and 5-year adjuvant tamoxifen

**Fig. 5:**
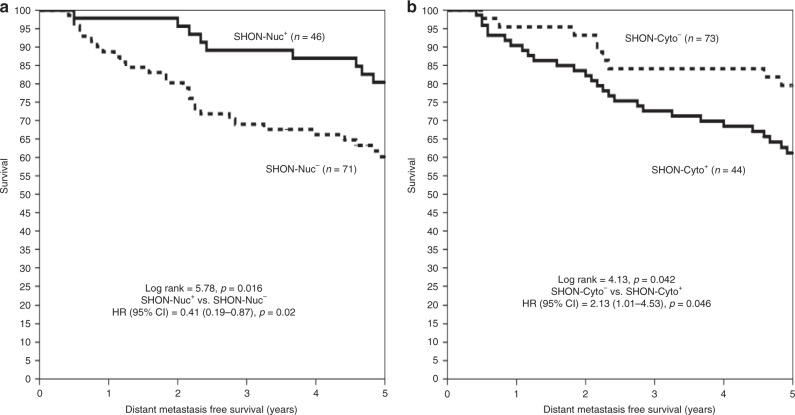
Clinical outcome of SHON protein nuclear and cytoplasmic expression in the chemotherapy-treated patients. Kaplan–Meier plots of the rates of distant metastasis-free survival (years) in the NUH-LABC cohort (*n* = 117), who received neoadjuvant anthracycline-based combination chemotherapy and if ERα^+^, followed by 5-year adjuvant tamoxifen, according to the status of SHON protein nuclear expression (SHON-Nuc) (**a**) and SHON protein cytoplasmic expression (SHON-Cyto) (**b**). The *p* value from the log rank test is shown in each panel; ‘*n*' is the number of samples in each group. + positive expression, − negative expression

In the NUH-LABC cohort, BC patients received the Neo-ACT chemotherapy followed by a 5-year adjuvant tamoxifen treatment if the tumours were ERα^+^. Patients with high nuclear SHON protein expression had a significantly lower distant relapse risk compared to low nuclear SHON protein expression [20 vs 39%; HR (95% CI) = 0.41 (0.19–0.87), *p* = 0.02] (Fig. 5a), whereas high SHON cytoplasmic expression had a significant higher distant relapse risk compared to low SHON cytoplasmic expression [44 vs 22%; HR (95% CI) = 2.13 (1.01–4.53), *p* = 0.046] (Fig. 5b). Moreover; a multivariate Cox regression model controlling for other validated prognostic factors and systemic therapy revealed that high cytoplasmic SHON expression was independently associated with a higher risk of distant relapse after the Neo-ACT and 5-year tamoxifen treatment [HR (95% CI) = 5.08 (1.13–44.52), *p* = 0.037]. The interaction term between ERα status and SHON nuclear expression was statistically significant in determining distant metastasis-free survival (*p* = 0.005). In addition, the interaction term between SHON nuclear expression and tamoxifen therapy was also highly significant (*p *= 0.007) (Table 3).

**Table 3:** Multivariate Cox regression model analyses for distant metastasis-free survival in the NUH-LABC cohort (*n* = 117)

Variables	OR	95% CI		*p* value
		Lower	Upper	
SHON cytoplasmic expression (high)	7.06	1.13	44.52	0.037^#^
Adjuvant tamoxifen endocrine therapy	0.01	0.001	0.11	0.061
ERα status	13.90	2.26	85.63	0.005^##^
Post chemotherapy lymph node status	0.999	0.995	1.003	0.697
Post chemotherapy lymph vascular invasion	1.003	0.999	1.007	0.090
Residual tumour size (mm)	1.002	0.998	1.005	0.287
Histological grade	0.807	0.390	1.673	0.565
HER2 status	1.020	0.414	2.513	0.966
ERα*SHON nuclear expression interaction				0.005^##^
ERα*SHON cytoplasmic expression Interaction				0.065
Adjuvant tamoxifen *SHON nuclear expression interaction				0.007^##^

*ERα* oestrogen receptor α, *HER2* human epidermal growth factor receptor 2

^#^*p* < 0.05, ^##^*p* < 0.01

### The relationship between SHON protein expression and response to Neo-ACT chemotherapy

We further investigated the association between SHON protein expression and the pathological complete response (pCR) in the NUH-LABC cohort, in which 117 patients had response data and 15% (17/117) achieved a pCR. SHON nuclear expression was detected in 39% (46/117) of the pre-chemotherapy core biopsies, whereas high cytoplasmic staining was observed in 62% (73/117) of the biopsies. No SHON expression was seen in 14.5% (17/117) of the biopsies, while 12% (14/117) showed both high nuclear and cytoplasmic staining, 50% (59/117) no nuclear but high cytoplasmic staining, and 23% (27/117) high nuclear but low cytoplasmic staining. Low SHON nuclear protein expression was associated with an increased proportion of patients achieving a pCR [21% (15/71) of the patients] compared with high SHON nuclear protein expression [4% (2/46) of the patients; OR (95% CI) = 5.88 (1.28–27.2203), *p* = 0.012]. High SHON cytoplasmic protein expression was associated with an increased proportion of patients achieving a pCR [21% (15/73) of the patients] compared with low SHON cytoplasmic protein expression [5% (2/44) of the patients; OR (95% CI) = 5.43 (1.18–25. 03), *p* = 0.017]. Multivariate logistic regression analyses showed that SHON high cytoplasmic staining, like SPAG5 overexpression,^10^ independently predicted the sensitivity to ACT (*i.e*., a higher pCR) [OR (95% CI) = 5.22 (1.03–26.47), *p *= 0.046] (Table 4)].

**Table 4:** Multivariate logistic regression model analyses for the pCR in the NUH-LABC cohort (*n* = 117)

Variables	OR	95% CI		*p* value
		Lower	Upper	
SHON cytoplasmic expression (high)	5.22	1.03	26.47	0.046*
ERα status (positive)	0.30	0.078	1.152	0.079
HER2 status (overexpression)	0.80	0.14	4.57	0.804
SPAG5 (overexpression)	4.84	1.274	18.36	0.021*

*ERα* oestrogen receptor α, *HER2* human epidermal growth factor receptor 2, *SPAG5* sperm-associated antigen 5

*p < 0.05

## Discussion

SHON is a recently identified novel secreted hominoid-specific oncoprotein in BC.^7^ We had previously generated a SHON polyclonal antibody and used it to perform IHC in the well-characterised Nottingham Tenovus primary breast carcinoma series.^9–11^ In that study, we demonstrated that SHON nuclear expression in breast tumours predicted the clinical outcome of patients who received tamoxifen in a high risk and ERα^+^ cohort.^7^ We have now developed a SHON monoclonal antibody and with it, we have not only validated our previous findings, but have also observed that SHON nuclear expression is actually an absolute determinant of survival outcomes with tamoxifen. Furthermore, we demonstrated that SHON cytoplasmic expression in ERα^−^ tumours predicted clinical outcomes in patients receiving anthracycline-based chemotherapy. Given that tamoxifen and chemotherapy resistance severely limits successful management of BC, SHON may serve as a biomarker for selection of patients for treatment in the clinic.

**Fig. 6:**
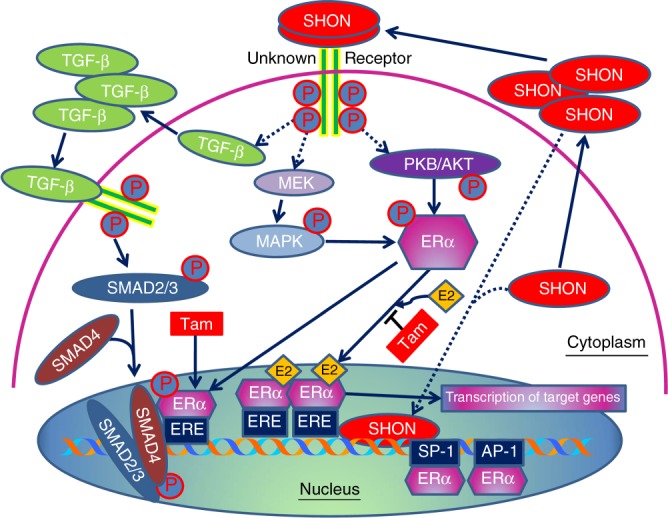
Current understanding of SHON and ERα signalling. Classically, ERα signalling is initiated following the binding of oestrogen (E2) to oestrogen receptor, resulting in its translocation to nucleus and binding directly to oestrogen response elements (EREs) on gene promoter of oestrogen-regulated genes, which subsequently activate transcription of downstream genes. Anti-oestrogen tamoxifen (Tam) competes with E2 for binding to ERα. SHON may bind to a yet-unknown receptor and activate PI3K/AKT and MAPK pathways that are linked to the action of ERα. SHON may also activate TGF-β pathway, resulting in SMAD2/3/4 translocation to nucleus and causing inhibition of ERα transcriptional activity upon Tam induction. Exogenous and/or intracellular pools of SHON may also enter the nucleus, thus enhancing the transcriptional activity of ERα

It is still unclear how SHON nuclear expression is able to impact on the efficacy of tamoxifen therapy. *SHON* is an oestrogen-regulated gene and the pure ERα antagonist ICI 182,780 partially attenuates SHON-stimulated growth promotion in MCF-7 breast cancer cells, indicating that SHON signalling is at least, in part, mediated by ERα.^7^ However, ERα-regulated functions are thought to play a pivotal role in determining the response to anti-oestrogen therapy. Several of the genes that the Oncotype DX test measures are ERα-regulated genes, including *PR*, *BCL-2* and *SCUBE2*.^15,16^ Therefore, ERα-driven genes may be of particular interest for the development of molecular biomarkers to predict response to endocrine treatment. It has been shown that forced expression of SHON increases phosphorylation of AKT and p^44/42^ MAPK and increases the expression of BCL-2 and NF-κB to mediate the oncogenicity of SHON.^7^ Therefore, SHON may modulate ERα signalling through the activation of p^44/42^ MAPK and PI3K/AKT/mTOR pathways and NF-κB transcriptional activation of BCL-2 (Fig. 6). SHON presumably functions in an autocrine/paracrine manner as other secreted growth factors. Secreted SHON may bind to and activate a yet-unknown cell surface receptor, which consequently activates the PI3K/AKT and MAPK pathways that are linked to the action of ERα, including transcription of target genes. Nuclear SHON may also be directly involved in oestrogen independent signalling of ERα, through modulation of the binding of ERα to other transcription factors *e.g*. SP-1 and AP-1. It has now been shown that many secreted growth factors, including prolactin, growth hormone, epidermal growth factor (EGF), interferon gamma and Schwannoma-derived growth factor, are located both in the cytoplasm and in the nucleus.^17^ Such differential subcellular localisations are often associated with distinctive functions. It is observed that some of these factors *e.g*. FGFs contain nuclear localisation signals, but others do not. In the case of FGF-1, it is the exogenous, rather than intracellular, pools of FGF-1 that enter the nucleus.^18,19^ Cytosolic accumulation and subsequent nuclear import of FGF-1 require PI3K signalling, and nuclear translocation of FGF-1 is dependent upon acidic vesicular pumps. Once in the nucleus, nuclear FGF-1 stimulates DNA synthesis, independent of cell surface signalling. Moreover, multiple growth factor receptors have also been found in the nucleus, including the prolactin receptor, growth hormone receptor and EGF receptors in the form of both intact and cleaved membrane associated receptors. ERα itself is a nuclear receptor. Therefore, it is possible that exogenous and/or intracellular pools of SHON may directly enter the nucleus, and thus enhance the transcriptional activity of ERα (Fig. 6). However, it is not yet clear how SHON enters the nucleus. Of note, SHON has also been shown to promote EMT through the TGF-β pathway via the mediation of SMAD2/3 signalling.^8^ Activated SMAD2/3 binds SMAD4 in cytoplasm, followed by the translocation of the SMAD2/3/4 complex into the nucleus to regulate the transcription of TGF-β-induced genes.^20,21^ Upon exposure to tamoxifen, SMAD4 binds ERα and serves as a transcription corepressor for ERα.^22,23^ Therefore, SHON nuclear expression could be a determinant of an active ERα signalling complex so that tamoxifen can effectively block ERα signalling. It is also possible that its nuclear localisation facilitates TGF-β-SMAD4 and ERα cross talk and inhibits ERα-mediated gene transcription (Fig. 6).

Biomarkers play a fundamental role in the personalisation of clinical breast cancer care for improved treatment outcomes. Despite more than a decade’s effort to develop new breast cancer biomarkers, only three biomarker tests (ERα, PR and HER2) are currently mandatory for those diagnosed with breast cancer.^24^ Other multigene tests are either useful only in a subgroup of breast cancers, including Oncotype DX, Prosigna, MammaPrint and EndoPredict, or simply investigational.^25^ They are commonly used to provide complementary prognostic information to clinicopathological features and predict chemotherapy benefit in early-stage hormone receptor-positive and HER2-negative BC.^26–28^ The development of multigene tests usually face issues such as insufficiently high levels of evidence, overfitting computational models and false discovery rates.^29^ In addition, they often do not yield significant improvement in predictive accuracy over the well-established pathological parameters such as histological grade.^30^ This is because these gene-expression biomarkers share common molecular pathways centred on cell proliferation and cell cycle regulation, which are the key components of the well-established pathological parameters.^30^ Moreover, MammaPrint and EndoPredic have been found to give different treatment recommendations for a portion of patients and cannot be used interchangeably,^31^ while Oncotype DX and MammaPrint offer different prognostic information to the same patients.^32^ Another issue with multigene tests is that some patients will still have an “intermediate” risk score, leading to an inconclusive prognosis,^26^ though chemotherapy may be surely spared in patients at intermediate recurrence scores as shown in the recent prospective TAILORx trail.^28^ Furthermore, although Oncotype DX can identify a group of patients with excellent prognosis when treated with adjuvant tamoxifen,^15,16^ it may provide no new biological insights into tamoxifen response than the simple measurement of ER and PR levels by the easy conventional IHC.^33^ It has now been demonstrated that a well selected single gene, such as SPAG5,^10^ ESPL1^34^ or Ki67,^35^ may be a better indicator of proliferation than the mixture of suboptimal proliferation genes included in the multigene tests.^36^ Such a protein biomarker would easily be implemented in the clinic as a routine test using conventional IHC techniques that have been used for ER at a fraction of the high cost associated with multigene tests.

In the current study, we also demonstrated that SHON cytoplasmic expression predicted better survival to adjuvant ACT chemotherapy in the ERα^−^ cohort, a higher pCR after receiving pre-operative ACT chemotherapy (chemotherapy responsiveness), and poor survival after 5-year tamoxifen treatment. In addition, SHON nuclear expression predicted favourable survival to adjuvant endocrine therapies, and a lower pCR after receiving pre-operative ACT chemotherapy (chemotherapy resistance). It is worthy of note that achieving a pCR after receiving neoadjuvant chemotherapy provides important prognostic information and is considered a surrogate endpoint for event-free survival in ERα^−^ or triple negative BC.^37–39^ In contrast, in ERα^+^ and HER2^+^ BC, the event-free survival is merely determined by the administration of targeting therapy: either endocrine or Herceptin therapy. Therefore, it was not surprising that SHON cytoplasmic expression was associated with a better survival outcome in our adjuvant ERα^−^ BC cohort whereas it was associated with poor survival in the neoadjuvant cohort (which was predominantly ERα^+^ BC) who received pre-operative chemotherapy followed by 5-year adjuvant tamoxifen although SHON cytoplasmic expression was associated with a higher pCR. Similarly, although SHON nuclear expression was associated with a lower pCR after receiving pre-operative chemotherapy, it was associated with better survival after 5-year tamoxifen therapy.

We previously demonstrated that SHON was also expressed in ERα^−^ BT549 and MDA-MB-231 BC cells.^7^ The current IHC analysis also showed that SHON cytoplasmic expression was significantly associated with aggressive BC phenotypes. Clinical data have previously indicated that as anti-oestrogen responsiveness increases, chemo-responsiveness decreases.^40,41^ We also showed that there was an inverse correlation between cytoplasmic and nuclear SHON expression in all the tumours. Therefore, it is consistent that nuclear SHON expression was linked to better survival to tamoxifen whereas cytoplasmic SHON expression was associated with better response to chemotherapy. High chromosomal instability and aneuploidy are hallmarks of malignant cells and confer vulnerability to chemotherapy.^42^ We demonstrated that SHON nuclear expression was highly associated with the expression of DNA repair proteins and a low proliferation index (Ki67), suggesting that SHON may be an important driver for genetic stability in BC, and SHON dysregulation could contribute to chromosomal instability. These findings are in agreement with previous studies that have suggested anthracycline works best in tumours with higher proliferation and chromosomal instability,^43,44^ whereas endocrine therapy is more effective in chromosomally stable, low proliferative BC.^45^

In summary, our study has clearly demonstrated that SHON expression in tumours is a potential biomarker for tamoxifen and chemotherapy responses, depending on its subcellular localisation. While SHON nuclear expression was able to predict patient outcomes to tamoxifen in ERα^+^ BC, SHON cytoplasmic expression could predict the response to ACT chemotherapy. However, the exact mechanism for its biomarker utility is still unclear. Identification of a potential SHON receptor, and determining the role of SHON in ERα^−^ BC cells will be the next priority in delineating its mechanisms of action. Multicentre prospective studies are required for confirmation and validation before SHON can be used as a clinical biomarker.

## Supplementary information

Supplementary Table S1

Supplementary Table S2

Supplementary Figure S1

## Data Availability

The data that support the findings of this study and materials described are available from the corresponding author upon reasonable request. Some restrictions may apply.
