# Supplementary material for: SHON expression predicts response and relapse risk of breast cancer patients after anthracycline-based combination chemotherapy or tamoxifen treatment
Source: Br J Cancer. 2019 Feb 28;120(7):728–45. doi: 10.1038/s41416-019-0405-x (PMC6461947; doi:10.1038/s41416-019-0405-x)
Supplement: Supplementary file 1 — Supplementary Table S1 [file 41416_2019_405_MOESM1_ESM.docx]

**Supplementary Table S1:** Clinicopathological characteristics of the patient demographics in all cohorts.

| **Variables**  **N (%)** | Nottingham University Hospital early stage primary BC cohort  N (%) | Nottingham University Hospital early stage ERα^-^ BC cohort  N (%) |
| --- | --- | --- |
| **Number of cases** | 1650 | 697 |
| **Age (months)**  Median, (Range), IQR | 53·6 (18-71), 31·5 | 51 (24-71) 16·6 |
| **No. of positive lymph nodes**  Negative  Positive  Unknown | 1056 (64·0)  594 (36·0)  0 (0·0) | 427 (61·3)  268 (38·5)  0 (0·0) |
| **Size (cm) - T stage**  T0  T1 a + b (≤10 mm)  T1 c (>10-20 mm)  T2 (>20-50 mm)  T3 (>50 mm)  T4 (tumour involves skin and/or the chest wall and/or is inflammatory)  Unknown | 0 (0·0)  187 (11·0)  868 (53·0)  579 (35·0)  16 (1·0)  0 (0·0)  0 (0·0) | 0 (0·0)  55 (7·9)  311 (44·6)  285 (40·9)  34 (4·9)  0 (0·0)  12 (1·7) |
| **Histological grade**  I  II  III  Unknown | 306 (18·5)  531 (32·2) 813 (49·3)  0 (0·0) | 10 (1·4)  67 (6·9)  619 (88·8)  1 (0·1) |
| **Estrogen-receptor status**  Positive  Negative  Unknown | 1211 (73·4)  439 (26·6)  0 (0·0) | 0 (0·0)  697 (100·0)  0 (0·0) |
| **Progestogen receptor status**  Positive  Negative  Unknown | 908 (55·0)  692 (42·0)  50 (3·0) | 11 (1·6)  625(89·7)  61 (8·8) |
| **HER2 overexpression**  No  Yes  Unknown | 1424 (86·3)  176 (10·7)  50 (3·0) | 530 (76·0)  153 (22·0)  14 (2·0) |
| **Treatment Type** | Adjuvant | Adjuvant |
| **Adjuvant Chemotherapy**  No  Yes  Unknown | 353 (21·4)  1297 (78·6)  0 (0·0) | 365 (52·1)  320 (45·9)  14 (2·0) |
| **Adjuvant Trastuzumab (Herceptin)**  No  Yes  Unknown | 1650 (100·0)  0 (0·0)  0 (0·0) | 685 (98·0)  0 (0·0)  14 (2·0) |
| **Adjuvant Hormonal therapy**  No  Yes  Unknown | 688 (41·7)  962 (58·3)  0 (0·0) | 685 (98·0)  0 (0·0)  14 (2·0) |
| **Any systemic therapy**  None  Hormone Therapy or Chemotherapy  Unknown | 655 (39·7)  995 (60·3)  0 (0·0) | 365 (52·1)  320 (45·9)  14 (2·0) |
| **Recurrence events**  No  Yes  Unknown | 995 (60·3)  627 (38·0)  28 (1·7) | 452 (64·8)  241 (34·6)  4 (0·6) |
| **Death events**  Alive, lost follow up or dead from other causes  Dead from breast cancer | 1262 (76·5)  388 (23·5) | 485 (68·6)  212 (30·4) |
| **Follow up (months)**  Median (Range), IQR | 143 (1-311), 60·0 | 113 (1-243), 81·28 |
| **MKI67 protein expression**  Low  High  unknown | 635 (38·5)  995 (60.3)  20 (1·2) | 105 (15·1)  526 (75.5)  66 (9·5) |
