# Supplementary material for: SHON expression predicts response and relapse risk of breast cancer patients after anthracycline-based combination chemotherapy or tamoxifen treatment
Source: Br J Cancer. 2019 Feb 28;120(7):728–45. doi: 10.1038/s41416-019-0405-x (PMC6461947; doi:10.1038/s41416-019-0405-x)
Supplement: Supplementary file 2 — Supplementary Table S2 [file 41416_2019_405_MOESM2_ESM.docx]

**Supplementary Table S2:** Antibodies and optimisation conditions used to immunohistochemically profile the Nottingham University Hospitals based cohorts. Detailed below are: antigens, primary antibodies, clone, source, optimal dilution and scoring system, used for each immunohistochemical marker.

| **Antigen** | **Antibody** | **Clone** | **Source** | **Antigen Retrieval** | **Dilution / Incubation Time** | **Distribution** | **Scoring**  **system** | **Cut-offs** |
| --- | --- | --- | --- | --- | --- | --- | --- | --- |
| p53 | Mouse MAb anti p53 | DO7 | Novocastra | Citrate pH6 | 1: 50  60 min | Nuclear | % of positive cells | ≤20% (negative)  >20% (High) |
| BCL2 | Mouse MAb anti-Bcl2 | 124 | Dako-Cytomation | Citrate pH6 | 1:100  60 min | Cytoplasm | % of positive cells | >10% (positive |
| BRCA1 | BRCA1 | MS110 | Calbiochem | Citrate pH6 | 1:100  60 min | Nuclear | % of positive cells | <25% (negative) |
| ATM | Rabbit MAb anti-ATM | Y170 | Abcam | Citrate pH6 | 1:100  18 hours | Nuclear | % of positive cells | <25% (negative) |
| p27 | anti-p27 | SX53G8 | Dako-Cytomation | Citrate pH6 | 1:50  60 min | Nuclear | % of positive cells | ≥10% (positive) |
| Vimentin | Mouse MAb anti-vimentin | Vim 3B4 | Dako-Cytomation | Citrate pH6 | 1:250  60 min | Cytoplasm | % of positive cells | ≥10% (positive) |
| Bax | Rabbit anti-Bax | Polyclonal | Abcam | Citrate pH6 | 1:1000  60 min | Cytoplasm | % positive cells | ≥10% (positive) |
| ER | Mouse MAb anti-ER-α | SP1 | Dako-Cytomation | Citrate pH6 | 1:150  30 min | Nuclear | Allred score | ≥3 (positive) |
| ER | Mouse MAb anti-ER-α | EP1 | Dako-Cytomation | Citrate pH6 | 1:80  30 min | Nuclear | % positive cells | ≥1% positive |
| PR | Mouse MAb anti-PR | PgR636 | Dako-Cytomation | Citrate pH6 | 1:125  30 min | Nuclear | % positive cells | ≥1% positive |
| EGFR | Mouse MAb anti-EGFR | 31G7 | Zymed Laboratories | Proteinase K, 370C for 8 min | 1:30  60 min | Membrane | 0-3 as HER2 | 0 or +1 (negative)  +2 or +3 (positive) |
| CK14 | Mouse MAb anti-Ck14 | LL002 | Novocastra | Citrate pH6 | 1:40  60 min | Cytoplasm | % of positive cells | ≥10% (positive) |
| Ck5/6 | Mouse MAb anti-Ck5/6 | D5/161B4 | Dako-Cytomation | EDTA pH8 | 1:100  60 min | Cytoplasm | % of positive cells | ≥10% (positive) |
| Ck17 | Mouse MAb anti-Ck17 | E3 | Dako-Cytomation | Citrate pH6 | 1:100  60 min | Cytoplasm | % of positive cells | ≥10% (positive) |
| Ck18 | Mouse MAb anti-Ck18 | DC10 | Dako-Cytomation | Citrate pH6 | 1:100  60 min | Cytoplasm | % of positive cells | ≥10% (positive) |
| HER2 | Rabbit antihuman c-erbB2 | polyclonal | Dako-Cytomation | None | 1:400  60 min | Membrane | See text | See text |
| Ki67 | Mouse MAb anti-Ki-67 | MIB1 | Dako-Cytomation | Citrate pH6 | 1:300  60 min | Nuclear | % of positive cells | 0-30% (low)  >30% (high) |
| TOP2A | Mouse MAb  TOP2A | KiS1 | Dako-Cytomation | Citrate pH6 | 1:100  60 min | Nuclear | % of positive cells | >25% (positive) |
| p21 | Mouse MAb anti-p21 | SW118 | Dako-Cytomation | Citrate pH6 | 1:50  60 min | Nuclear | % of positive cells | ≥10% (positive) |
| MDM2 | Mouse MAb anti-MDM2 | 1B10 | Novocastra | Citrate pH6 | 1:200  60 min | Nuclear | % of positive cells | ≥10% (High) |
| MDM4 | Affinity purified rabbit anti-HdmX/MDM4 | IHC-00108 | Bethyl Labs | Citrate pH6 | 1:100  60 min | Nuclear | % of positive cells | 0-20% (Low)  >20% (High) |
| HER3 | Mouse MAb anti-HER3 | RTJ1 | Novocastra | Citrate pH6 | 1:20  60 min | Cytoplasm and Membrane | H score | H score <150 |
| HER4 | Rabbit antihuman c-erbB4 | polyclonal | Neo Marker | None | 6:4  60 min | Cytoplasm | H score | H score <100 |
| P-cadherin | Mouse MAb anti-P-cadherin | Clone 56 | BD Bioscience | None | 1:200  60 min | cytoplasm | % of positive cells | >5% positive |
| E-cadherin | Mouse MAb anti-E-cadherin | HERCD-1 | Zymed Laboratories | Citrate pH6 | 1:100  60 min | Membrane | H score | H score ≤100 |
| SPAG5 | Rabbit anti-SPAG5 | polyclonal | Sigma-Aldrich | Citrate pH6 | 1:50  60 min | Cytoplasmic | % of positive cells | >10% (positive) |

All sections were pre-treated with microwave antigen retrieval using 0·1% citrate buffer (pH 6) except for HER2 (no pre-treatment) and EGFR (pre-treated with protease for 10 minutes). MAb: Monoclonal antibody; MDM2: murine double minute 2; MDM4: murine double minute 4; ATM: ataxia telangiectasia mutated; BRCA1: BC 1, ER: oestrogen receptor; PR: progesterone receptor; CK: cytokeratin; EGFR: epidermal growth factor; TOP2A: Topoisomerase II alpha; HAGE: helicase Antigen, HER2 (ERBB2): v-erb-b2 erythroblastic leukemia viral oncogene homolog 2, neuro/glioblastoma derived oncogene homolog (avian), HER3 (ERBB3): v-erb-b2 erythroblastic leukemia viral oncogene homolog 3 (avian), HER4 (ERBB4): human epidermal receptor 4 , BCL2: B-cell CLL/lymphoma 2, Bax: BCL2-associated X protein , SPAG5: sperm associated antigen 5, KIF2C: kinesin family member 2C.
