# Supplementary material for: SHON expression predicts response and relapse risk of breast cancer patients after anthracycline-based combination chemotherapy or tamoxifen treatment
Source: Br J Cancer. 2019 Feb 28;120(7):728–45. doi: 10.1038/s41416-019-0405-x (PMC6461947; doi:10.1038/s41416-019-0405-x)
Supplement: Supplementary file 3 — Supplementary Figure S1 [file 41416_2019_405_MOESM3_ESM.docx]

**Supplementary Figure S1**

**Specificity of the mouse anti-SHON monoclonal antibody (mAb)**

The anti-SHON mAb was able to specifically recognise both the endogenous and forced expression of SHON protein in human breast cancer MCF-7 cells by western blot analysis (**Fig. S1A** left panel). As we reported previously using a rabbit anti-SHON polyclonal antibody ^[1]^, the expression of SHON protein in human normal breast MCF10A cells was not detected while forced SHON expression was evidently detectible (**Fig. S1A** right panel). In addition, the specificity of the mouse anti-SHON mAb was further demonstrated in a couple of cancer cell lines by western blot (**Fig. S1B**) and by indirect immunofluorescence staining (**Fig. S1C).**

| **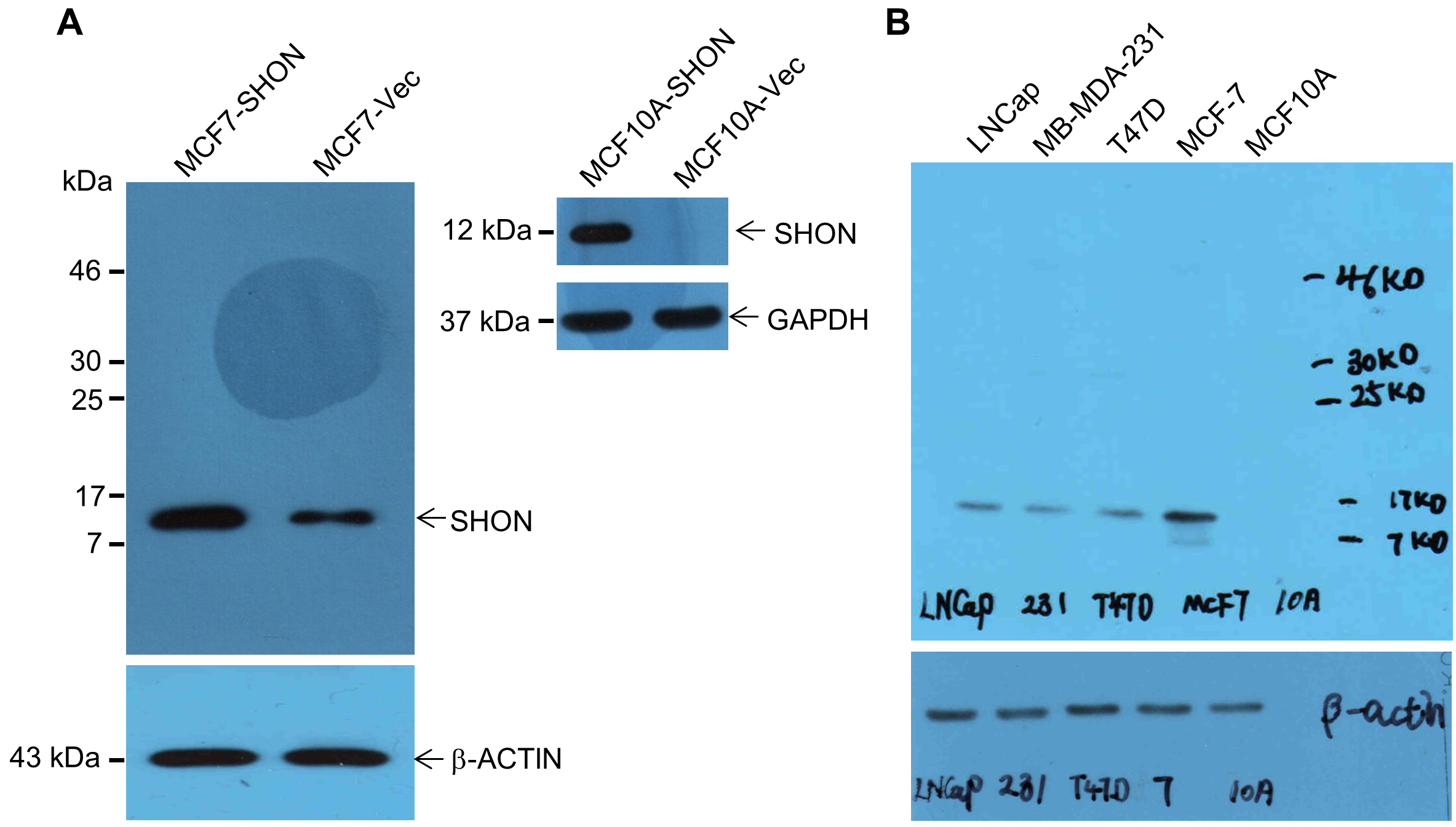**  **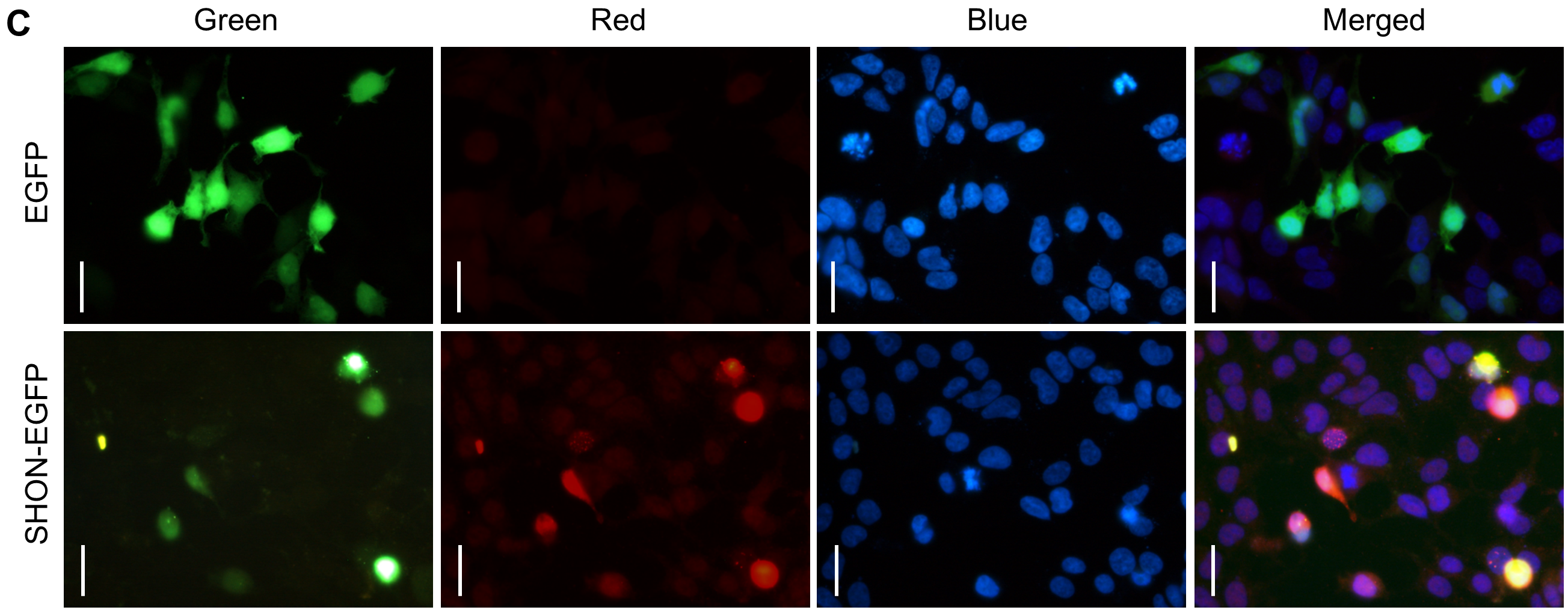**  **Fig. S1. Specificity of the mouse anti-SHON monoclonal antibody**. (**A**) Western blot of stably transfected cell lines. Human breast cancer MCF-7 (left panel) and normal breast MCF10A (right panel) cell lines were stably transfected with the SHON expression plasmid pIRESneo3-SHON (*SHON*) and the empty control pIRESneo3 vector (*Vec*). Whole cell lysates of the stable cells: MCF7-Vec and MCF7-SHON, and MCF10A-Vec and MCF10A-SHON were immuno-blotted with the affinity purified mouse anti-SHON monoclonal antibody. β-actin or GAPDH protein expression was used as the cell lysate input control. (**B**) SHON protein expression in several human breast cell lines (MDA-MB-231, T47D and MCF-7) and the MCF10A normal breast cell line, as well as the human prostate cancer cell line LNCap, were detected by Western blot using the affinity purified mouse anti-SHON monoclonal antibody. (**C**) HEK293 cells were transiently transfected with the expression plasmid pIRESneo3-SHON-EGFP, which encodes SHON protein with a C-terminal EGFP tag (*SHON-EGFP*), and the pEGFP-C1 empty vector, which encodes the EGFP protein (*EGFP*). 24h post-transfection, cells were fixed and permeabilized with Triton X-100 for immunocytochemical staining with the affinity purified mouse anti-SHON monoclonal antibody as the primary antibody. The staining of SHON was then visualized with a Cy5 cyanine dye conjugated secondary antibody (*Red*) and counterstained with Hoechst 33258 (*Blue*). The expression of EGFP or SHON-EGFP (*Green*) was examined by fluorescence microscopy. Merged images are shown in the far right column. The co-localization of green and red fluorescence staining in the pIRESneo3-SHON-EGFP transfected cells demonstrated that the mouse anti-SHON monoclonal antibody specifically recognized SHON protein. Molecular weights of detected protein bands in kDa are shown on the sides. Bar, 50 µm. |
| --- |

**References**

[1] Jung Y, Abdel-Fatah TM, Chan SY, Nolan CC, Green AR, Ellis IO, Li L, Huang B, Lu J, Xu B, Chen L, Ma RZ, Zhang M, Wang J, Wu Z, Zhu T, Perry JK, Lobie PE, Liu DX**.** SHON is a novel estrogen-regulated oncogene in mammary carcinoma that predicts patient response to endocrine therapy. *Cancer Res*. 2013; 73:6951-6962
